# Metabolic Dysregulation in Parkinson’s Disease: Non-Oxidative Phosphorylation and Its Role in Brain Energy Metabolism

**DOI:** 10.14336/AD.2025.0619

**Published:** 2025-06-22

**Authors:** Marta Pokotylo, Norbert Brüggemann, Jannik Prasuhn

**Affiliations:** ^1^Department of Neurology, University of Medical Center Schleswig-Holstein, Campus Lübeck, Lübeck, Germany.; ^2^Center for Brain, Behaviour, and Metabolism, University of Lübeck, Lübeck, Germany.; ^3^Institute of Neurogenetics, University of Lübeck, Lübeck, Germany.; ^4^Department of Neurology, Johns Hopkins University School of Medicine, Baltimore, Maryland, USA.; ^5^F.M. Kirby Research Center for Functional Brain Imaging, Kennedy Krieger Institute, Baltimore, Maryland, USA.

**Keywords:** Parkinson’s disease;, mitochondrial dysfunction, glycolysis, TCA cycle, magnetic resonance spectroscopy imaging

## Abstract

Parkinson’s disease (PD) is a progressive neurodegenerative condition affecting around 1-2% of the population over the age of 60. The lack of disease-modifying therapies highlights the need for insights into the etiology and pathogenesis of PD. Mitochondrial dysfunction is recognized to be a significant contributor to disease pathogenesis, resulting in bioenergetic deficits and subsequent neurodegeneration. Research indicates that changes in non-oxidative phosphorylation (non-OXPHOS) metabolism in PD may serve as an adaptive response to mitochondrial dysfunction, compensating for energetic failure and alleviating disease progression. This review explores mitochondrial dysfunction-driven alterations in non-OXPHOS metabolic pathways, such as glycolysis and the tricarboxylic acid cycle, emphasizing their role in maintaining energy metabolism and their dual contribution to neuroprotection and disease progression. Advances in neuroimaging techniques are also discussed, particularly their role in visualizing metabolic changes *in vivo* and their potential utility in identifying non-OXPHOS metabolism as a biomarker of mitochondrial dysfunction. By enhancing our understanding of the complex interplay between metabolic pathways in PD, this review underscores the importance of personalized therapeutic approaches that consider individual metabolic variations. Ultimately, these insights aim to pave the way for improved diagnostic utility and personalized treatment strategies that address the metabolic and mitochondrial dysfunctions underlying PD pathogenesis.

## 1. Introduction

Parkinson’s Disease (PD) is one of the most prevalent neurodegenerative diseases, affecting approximately 1-2% of the population aged over 60 years [1]. The clinical presentation of PD includes motor deficits like tremors, bradykinesia, and rigidity, along with non-motor manifestations, such as gastrointestinal and autonomic symptoms, sleep disturbances, and dementia [2]. Around 15% of PD cases are attributed to a known genetic cause related to the strong genetic risk factor GBA1 (~10%), or pathogenic mutations in *SNCA, LRRK2, PRKN, PARK7 (DJ-1), PINK1*, and some more rare genes while 85% remain without a known underlying monogenic cause [3,4]. The PD primary etiology is characterized by the accumulation of Lewy bodies composed of alpha-synuclein (aSyn) fibrils and the subsequent loss of dopaminergic neurons in the *substantia nigra pars compacta* (SNpc) [5]. Mitochondrial dysfunction is recognized to play a crucial role in PD pathogenesis, affecting various aspects of mitochondrial homeostasis, including adenosine triphosphate (ATP) production via oxidative phosphorylation (OXPHOS), iron and calcium homeostasis, and mitigation of oxidative stress [6-8]. The evidence of mitochondrial dysfunction derived from environmental, genetic, *in vitro*, and *in vivo* studies is substantial, supporting the idea that mitochondrial dysfunction may be the primary driver of PD pathogenesis, at least in a subfraction of patients [9-12].

**Figure 1.**
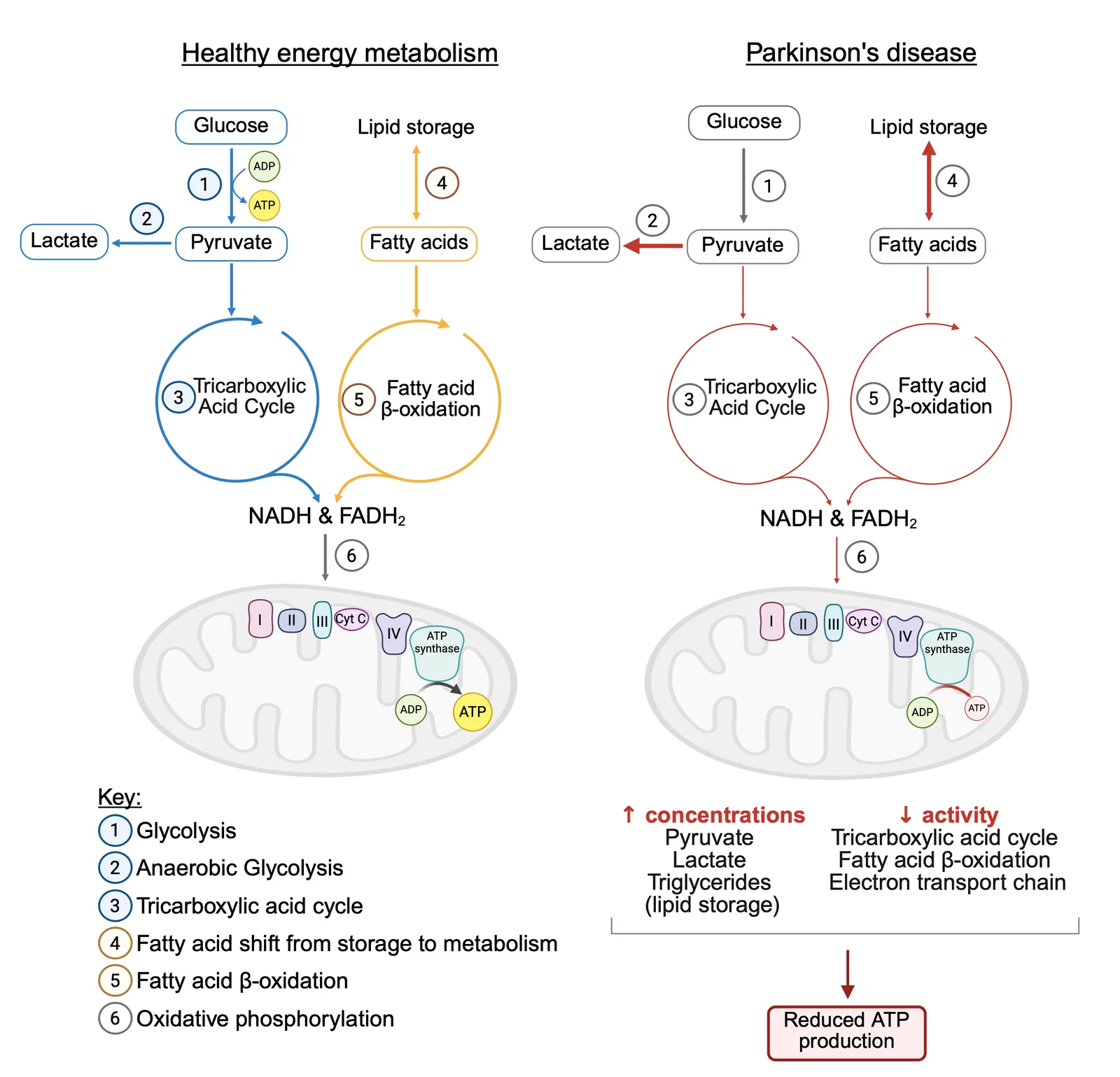
**Physiological energy metabolism compared to Parkinson’s disease pathophysiology**. This schematic representation compares the metabolic states under healthy conditions and in the Parkinson’s disease (PD) context. The left panel depicts healthy energy metabolism comprising glucose (blue arrows) and lipid metabolism (yellow arrows). Glucose is metabolized into pyruvate via glycolysis, which enters the tricarboxylic acid (TCA) cycle. Depending on the cellular energy demands, pyruvate can be converted into lactate in anaerobic glycolysis. Parallelly, fatty acids are diverted from storage towards metabolism and undergo fatty acid β-oxidation (FAO). TCA cycle and FAO function to generate reduced forms of nicotinamide adenine dinucleotide (NADH) and flavin adenine nucleotide (FADH_2_), which drive oxidative phosphorylation taking place in the mitochondria, resulting in adenosine triphosphate (ATP) production. The right panel represents metabolic dysregulation in PD, where key metabolic processes are disrupted. The red color of the arrows signifies metabolic alterations in PD, including presentation of elevated levels of lactate, pyruvate and triglycerides with thicker arrows, suggesting increased lipid storage and anaerobic glycolysis. Whilst the activity of the TCA cycle, FAO and electron transport chain have been reported to be reduced, and is annotated by thinner arrows. Collectively, these metabolic dysregulations result in reduced ATP production, exacerbating neurodegeneration and disease progression. Abbreviations list: ADP: adenosine diphosphate; ATP: adenosine triphosphate; CoQ: coenzyme Q; Cyt C: cytochrome C; FADH_2_: flavin adenine nucleotide (reduced form); FAO - fatty acid β-oxidation; NADH: nicotinamide adenine dinucleotide (reduced form); PD: Parkinson’s disease; TCA: tricarboxylic acid. Figure created using BioRender.com

The brain is a highly metabolically demanding organ, consuming 5.9 mg of glucose per 100 mg of brain tissue per minute, corresponding to 20% of the calories available to the entire body [13,14]. Under healthy conditions, the necessary energy is supplied by the oxidative metabolism of glucose via OXPHOS in the mitochondrial electron transport chain (ETC), facilitating the maintenance of synaptic transmission and neuronal function [15]. Extensive postmortem and *in vivo* studies provide compelling evidence of ETC protein complex impairments among patients with PD (PwPD) [16-18]. Such dysfunctions reduce ATP synthase activity and ATP production, triggering a metabolic switch to a non-OXPHOS pathway to meet neuronal energy demands (Fig. 1) [19]. However, energy metabolism involves a complex interplay of various metabolic pathways, and the adaptive changes occurring in PD, as well as their consequences and benefits, are not fully understood.

An emerging idea suggests that PD is a metabolic disorder, the notion supported by epidemiological studies demonstrating an association between metabolic disorders such as type 2 diabetes mellitus (T2DM) and PD [20,21]. Chronic inflammation, insulin signaling dysregulation, mitochondrial dysfunction, and impaired glucose metabolism are some of the shared pathophysiological mechanisms, suggesting that T2DM pathogenesis may predispose to PD development through overlapping disease mechanisms [20]. While the association of T2DM and PD is beyond the scope of this review, their high prevalence highlights the necessity of investigating metabolic alterations in PD to better understand its mechanism, improve diagnostic capabilities, and develop improved therapeutic options.

This review investigates mitochondrial dysfunction-driven alterations in non-OXPHOS metabolic pathways in PD, including glycolysis, the tricarboxylic acid (TCA) cycle, and fatty acid β-oxidation (FAO), examining the profound implications of these disruptions for disease progression. The current literature on the compensatory mechanisms and neurotoxic consequences of brain non-OXPHOS metabolism alterations seen in PD will be discussed. In light of available research, the clinical relevance of this research topic will be highlighted, including the therapeutic opportunities for targeting and modulating non-OXPHOS metabolism, evaluating its capacity to affect PD progression. The lack of *in vivo* biomarkers for mitochondrial dysfunction in PD remains a crucial research gap. Therefore, this review explores the potential of non-OXPHOS metabolic pathways to serve this function alongside *in vivo* methods for their investigation.

## 2. Glycolysis

Besides neurons, the brain comprises astrocytes and microglia, which collectively exhibit lower rates of oxidative metabolism and energy consumption than neurons [22]. Notably, these cells possess distinct metabolic profiles and preferences; neurons have higher carbon dioxide production, consistent with OXPHOS functioning, whereas astrocytes present an enzyme profile consistent with glycolytic activity [23]. The cellular metabolic profile depends on the enzyme 2-kinase/fructose-2,6-bisphosphate-3 (PFKFB3), which regulates the degradation of fructose-2,6-phosphate and acts as a glycolytic activator [23]. Neuronal PFKFB3 is post-translationally downregulated, causing its continual degradation via the ubiquitin-proteasome pathway, particularly by E3 ubiquitin ligase [24]. PFKFB3 degradation in neurons results in glycolysis downregulation and redirection of glucose towards the pentose phosphate pathway, while adenosine monophosphate-activated protein kinase (AMPK) signaling activates astrocytic PFKFB3, promoting glycolysis [25]. Glycolysis is a metabolic process that converts one glucose molecule into two molecules of pyruvate and ATP. Although glycolysis is a less efficient ATP producer compared to OXPHOS, it operates at a faster rate, making it suitable for supporting acute neuronal energy demands [26]. Astrocytic upregulation of glycolysis in response to pathological conditions contrasts with the neuronal limited capacity. Nevertheless, neurons readily adapt to OXPHOS impairment by increasing the glycolytic rate to meet energy demands - a well-documented phenomenon observed in various *in vitro*, *in vivo*, and *ex vivo* PD models [27-39].

**Figure 2.**
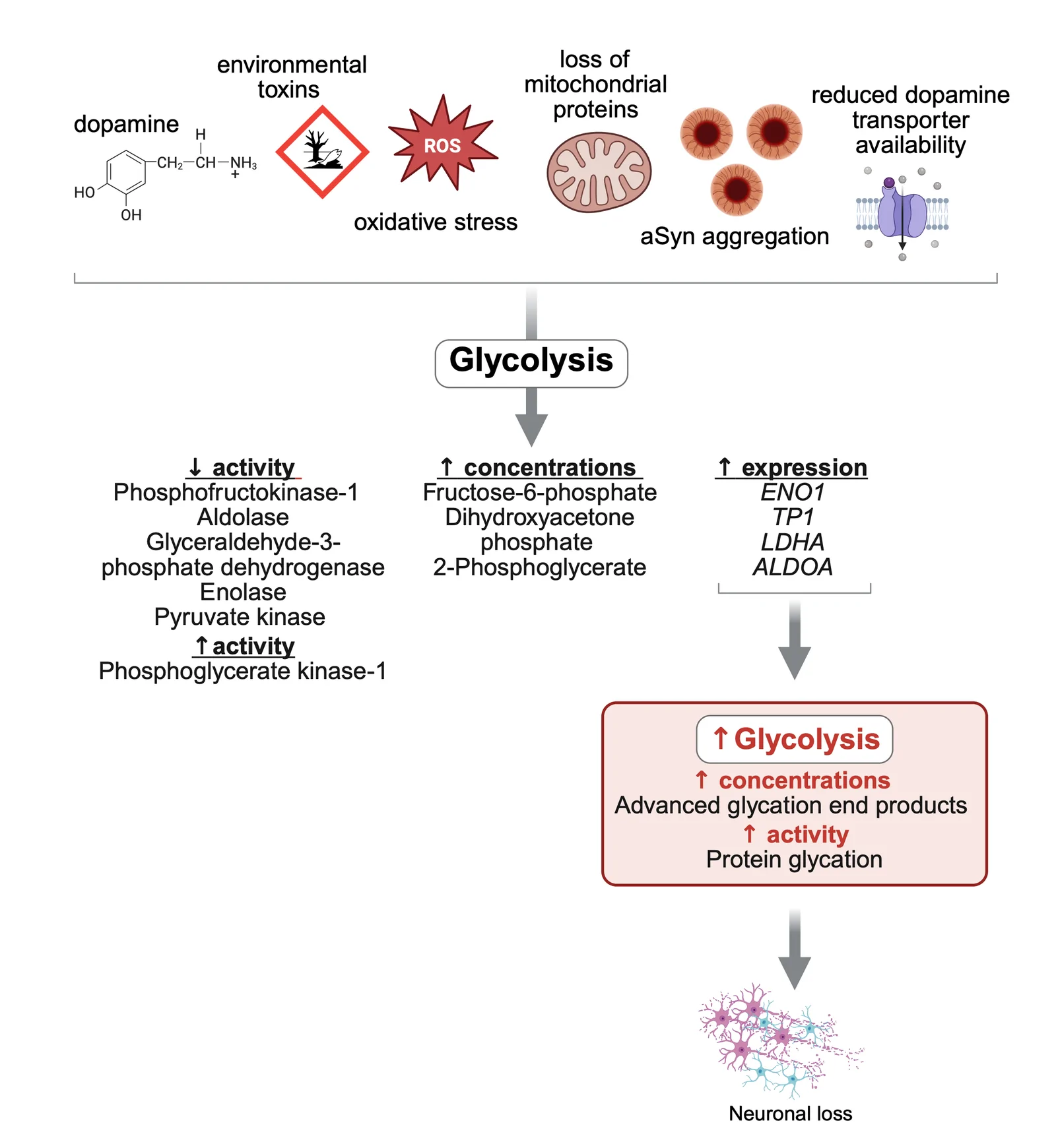
**Glycolysis dysregulation in Parkinson’s disease**. The glycolysis is modulated in Parkinson’ s disease (PD) in response to multiple pathogenic factors, ultimately exacerbating neurodegeneration. Some of the PD-related pathogenic factors such as, dopamine binding, environmental factors such as compounds in tire wear, loss of mitochondrial proteins, alpha-synuclein (aSyn) aggregation, and reduced availability of dopamine transporters affect glycolysis, driving its reprogramming. Numerous *in vitro* and *in vivo* studies identified altered activity of glycolytic enzymes in PD, with some reported to have increased activity such as aldolase and enolase, while phosphoglycerate exhibited reduced activity. Conversely, alterations in glycolytic metabolites have been identified, specifically reporting higher concentrations of fructose-6-phosphate, dihydroxyacetone phosphate, and 2-phosphoglycerate. In addition, the expression of glycolysis related genes such as *ENO1, TP1, LDHA,* and *ALDOA,* was found to be higher in PD [27-39]. The reported glycolytic changes collectively upregulate glycolytic activity, resulting in increased concentrations of advanced glycation end products. As supported by extensive research, increased protein glycation is one of the outcomes, that triggers mitochondria-induced apoptosis and heightened oxidative stress, further worsening neuronal loss [60-65]. Abbreviations list: aSyn: alpha-synuclein; PD: Parkinson’s disease. Figure created using BioRender.com

Phosphoglycerate kinase 1 (PGK1) is one of the key glycolytic enzymes that has garnered extensive research attention. PGK1 deficiency has been linked to early-onset parkinsonism, suggesting that glycolytic impairments contribute to the disease’s development and progression [40,41]. This discovery was followed by a paper detailing patients of juvenile-onset, levodopa-responsive parkinsonism with evidence of nigrostriatal dysfunction attributed to PGK1 deficiency, cementing glycolysis’ involvement in PD [42]. Enhanced PGK1 activity mitigates the loss of dopaminergic neurons, reduces PD-related symptoms, and slows disease progression in genetic-, chemical models of PD, and in induced pluripotent stem cells (iPSCs), highlighting the benefits of increased glycolysis in PD [43]. A new parkinsonism model targeting *Drosophila Pgk* results in locomotor deficits and progressive dopaminergic neuronal loss with ageing, supporting the link between glycolytic activity and PD [44]. Analysis of red blood cells from PwPD revealed elevated PGK1 levels, negatively correlated with the binding ratio of the dopamine transporter, suggesting a complex compensatory role of PGK1 in PD pathogenesis, which requires further investigation (Fig. 2) [27].

iPSC-derived dopaminergic neurons with *SNCA*^A53Y^ mutations exhibited upregulated glycolysis-related genes *LDHA*, *ENO1*, *TPI1*, and *ALDOA* upon oxidative stress exposure [28]. Additionally, high concentrations of an environmental toxin, N-(1,2-dimethylbutyl)-N’-phenyl-p-phenylenediamine quinone (6PPD-Q) have been identified in the cerebrospinal fluid (CSF) of PwPD. The exposure of primary dopaminergic neurons to 6PPD-Q reduced glycolytic metabolites, including fructose 6-phosphate, dihydroxyacetone phosphate, 2-phosphoglycerate, and pyruvate, alongside exacerbating Lewy body formation [45]. The loss of *DJ-1β* in a *Drosophila* model resulted in increased activity of phosphofructokinase-1 (PFK-1), enolase, and pyruvate kinase (PK), indicating metabolic reprogramming as a consequence of the loss of the PD-related gene [29]. In the search for metabolic markers of PD, proton magnetic resonance spectroscopy imaging (H^1^-MRSI) revealed significantly increased lactate levels in the prodromal stage of PD, while pyruvate concentration rose with disease progression [46]. This finding solidifies the idea of increased reliance on glycolysis as a result of mitochondrial dysfunction to compensate for cellular energy deficits.

A wealth of evidence supports the role of increased glycolysis in mitigating aspects of PD pathophysiology and phenotype. For instance, uric acid’s antioxidant properties enhance self-renewal and differentiation potential via increased hexokinase (HK), PFKFB3, aldolase, and lactate dehydrogenase (LDH) expression [47]. Similarly, zaprinast application in the *DJ-1* mutant *Drosophila* model improved PD-related phenotypes by increasing HX and enolase activity [48]. Erythropoietin and deferoxamine have demonstrated similar neuroprotective effects [49,50]. Polydatin shows promise in rescuing dopaminergic neurodegeneration and restoring motor function by boosting pyruvate levels and ATP production [51]. The protective effects of hydrogen sulfide have been attributed to enhanced Warburg effect activity via increased pyruvate dehydrogenase (PDH) activity [52]. A meta-analysis of 678,433 participants revealed a 20% reduction in PD incidences among participants treated with glycolysis enhancers such as PGK1a enhancers [53]. This research sparked an interest in the ‘terazosin’, an alpha-1-adrenergic receptor blocker used to treat prostatic hyperplasia and essential hypertension [54]. The beneficial effects of terazosin in PD are attributed to its ability to increase the glycolytic rate, specifically enhancing the activity of PGK1 [55]. Terazosin has shown neuroprotective effects in rodent PD models, slowing disease progression, improving cognitive dysfunction, and decreasing the frequency of PD-related diagnoses [55,56]. A pioneering study investigated the target engagement of terazosin in PwPD (n=13), demonstrating increased βATP/inorganic phosphate measured by phosphorus magnetic resonance spectroscopy imaging (^31^P-MRSI), validating the concept of enhancing glycolysis as a possible treatment [57]. Despite the promising findings, participants reported dizziness/lightheadedness, classified as mild side effects [57]. The reported mild side effects can be attributed to off-target effects, as terazosin binds to α_1_-adrenergic receptors, which are widely distributed in the brainstem [58]. As automatic dysfunction is predominant in PwPD, even in the early stages of the disease, some patients may be highly susceptible to the off-target effects of terazosin, highlighting the need for patient stratification by means of clinical presentation [59].

However, glycolysis upregulation has limitations, including the accumulation of toxic intermediates such as 1,3-bisphosphoglycerate and methylglyoxal (MGO), which contribute to protein and lipid modifications, aSyn aggregation, and glycogen-like damage (Figure 2) [60-62]. A single dose of MGO in aSyn transgenic mice increased protein glycation, accelerating motor and cognitive declines associated with PD, highlighting the toxic role of MGO in PD pathogenesis [63]. Furthermore, MGO is a precursor for tetrahydroisoquinoline (ADTIQ), a dopamine-related toxin that triggers mitochondrial apoptosis in dopaminergic neurons [64]. Increased glycolytic rates result in the accumulation of advanced glycation end products (AGEs), such as pentosidine, which is associated with lower cognition in PwPD, possibly contributing to PD pathogenesis [65]. Additionally, the accumulation of AGEs and associated protein glycation damage was observed in *DJ-1^-/-^* human midbrain organoids, highlighting the damaging consequences of excessive glycolysis [66].

The majority of evidence points toward upregulated glycolysis as a neuroprotective mechanism in PD; however, conflicting evidence suggests that reduction of specific glycolytic steps’ activity may also be beneficial. For instance, evidence of glyceraldehyde-3-phosphate dehydrogenase (GAPDH) co-localization with aSyn aggregates in post-mortem PD tissue, which inhibits GAPDH activity and reduces the rate of downstream glycolytic steps [67,68]. Therefore, GAPDH inhibition may serve as an adaptive response, offering neuroprotective effects such as the alleviation of aSyn aggregation in human neuroblastoma cells [69]. However, it’s important to consider the possibility of different glycolytic steps being affected to varying degrees across the disease span, as a reduction or inhibition of one glycolytic step does not implicate a decrease in overall glycolytic rate.

Nevertheless, evidence of cerebral glucose hypometabolism in PwPD suggests that neurons fail to maintain sufficient energy production, exacerbating the ATP deficiency and resulting in metabolic impairment [70-72]. Glycolytic failure is driven by multiple factors that become increasingly complex with disease progression. For instance, nicotinamide adenine dinucleotide (NAD^+^) metabolism, crucial for glycolysis, relies on its oxidation in the ETC for regeneration. Magnetic resonance spectroscopy imaging (MRSI) revealed reduced NAD^+^ levels in PwPD, indicating NAD^+^ metabolism dysregulation [73]. Nicotinamide riboside, a NAD^+^ precursor application, increased PK expression in 1-methyl-4-phenyl-1,2,3,6-tetrahydropyridine (MPTP)-treated zebrafish, improving survival time, reducing motor dysfunction, and alleviating endoplasmic reticulum stress [74]. Furthermore, NAD^+^ replenishment therapy improves mild cognitive impairments and induces transcriptional upregulation of mitochondrial processes in PwPD [75]. Impaired NAD^+^ metabolism results in a cycle of energy depletion, causing temporary glycolysis compensation until its unsustainability, leading to a metabolic bottleneck and exacerbation of the energy deficiency in PD. Another contributing factor is symptomatic PD treatment, such as Levodopa. Investigation into dopamine interactions revealed its capacity to bind enolase, aldolase, and PK, inhibiting their activity, worsening cellular energy state, and underlying motor complications such as dyskinesia associated with long-term Levodopa use [76]. With PD progression, multiple factors interact to influence glycolytic activity, complicating the task of differentiating between individual contributors to glycolytic dysregulation.

Mitochondrial dysfunction in PD drives metabolic reprogramming, initiating the glycolytic modulation, which temporarily offsets energy deficits but ultimately fails to compensate for OXPHOS dysfunction, exacerbating progressive metabolic failure. The limited understanding of the glycolytic switch in various brain cells, including astrocytes and microglia, warrants further research to gain mechanistic insights, determine its role in disease progression, and therapeutic potential. The evidence of glycolysis’ role in PD is conflicting, demonstrating the upregulation of specific glycolytic enzymes as a neuroprotective mechanism, while increased glycolytic flux results in the accumulation of toxic end-products, exacerbating neurodegeneration. The dual nature of glycolysis necessitates identifying the threshold balancing the compensatory benefits against neurotoxic effects. The lack of consistency suggests that the glycolytic reprogramming in PD is context-dependent, with factors such as cell type, disease stage, underlying genotype, and threshold of glycolytic flux shaping its contribution to disease progression. Currently, no research readily addresses the relationship between metabolic reprogramming and disease stage, due to challenges such as additional pathogenic factors coming into play, such as oxidative stress and aSyn aggregation with disease progression. Additional pathogenetic events complicate the procedure of assigning neurodegenerative processes to just one specific pathogenic process, reflecting the complex nature of PD. However, this issue could be partially addressed by adhering to a longitudinal study design, utilizing iPSC-derived neuron-glia co-cultures or brain organoids, alongside *in vivo* approaches such as MRSI to trace metabolites of interest and glycolytic flux to understand metabolic shifts along disease progression. Such understanding would provide the means for PD progression monitoring and explain the environment and conditions under which glycolysis adopts neuroprotective or neurotoxic roles. Furthermore, these insights would guide patient stratification and personalized therapeutic strategy development, informed by the patient’s metabolic profile, enhancing its efficacy while reducing side effects. Despite extensive gaps in our understanding, sufficient evidence already supports the therapeutic potential of glycolysis manipulation, including the successful use of NAD^+^ replenishment therapy and PGK1a activators in preclinical models and PwPD. The repurposing of terazosin has provided valuable evidence of glycolytic modulation efficacy in PwPD, yet demonstrated mild off-target effects in the context of PD. This research demonstrates that drug repurposing serves as a good starting point and a valuable method for proof of concept; however, its findings should be used to inform therapeutics development, specifically designed to address glycolysis modulation in PwPD, with minimal side effects and patient burden.

## 3. Lactate metabolism

Astroglia are highly glycolytic, with their energy expenditure not equating to energy production [77]. Astroglia utilizes glucose for lactate generation, directed towards its subsequent transport to neurons as an energy substrate, particularly when glycolysis upregulation is not sufficient [78,79]. Therefore, mitochondrial dysfunction-driven energy deficiency in neurons may increase astrocytic lactate production in PD; however, this requires further investigation. The astrocyte-neuronal lactate shuttle (ANLS) model has been proposed to elucidate the transfer of lactate between astrocytes and neurons [80]. The ANLS model involves the transport of lactate into the intracellular space via monocarboxylate transporter (MCT)1 and MCT4, followed by the neuronal uptake via MCT2. Within neurons, lactate is converted into pyruvate and directed into the TCA cycle to fuel ATP production [81].

The metabolic switch in PD is believed to shift specifically towards anaerobic glycolysis in a phenomenon known as the “neuronal Warburg effect”, first described in cancerous cells [82,83]. Some reinforcing evidence includes the upregulation of LDH in the *SNCA*-*Drosophila* model and elevated expression of HK and LDH in chemically induced PD *in vitro* and *in vivo* models [49,84]. LDH upregulation, in turn, prompts dopaminergic degeneration via increased AMPK activity and mammalian target of rapamycin suppression [84]. Similarly, *PINK^-1/-1^ Drosophila* demonstrates increased LDH and decreased ETC activity, further highlighting the metabolic shift in the PD model [30]. Elevated lactate levels in the CSF of PwPD have been linked to disease progression and neurodegeneration markers [85,86]. Increased lactate levels may impact PD pathogenesis; however, the complex interplay of biological processes, including the involvement of PK in lactate regulation, already implicated in PD pathogenesis, remains poorly understood [87].

The role of lactate in PD pathogenesis remains highly debatable, with no clear consensus on its benefits or drawbacks. Evidence suggests that lactate enhances mitochondrial function by lowering cellular pH to non-toxic levels, thereby activating mitophagy and autophagy in the 1-methyl-4-phenylpyridinium (MPP^+^)-treated mouse model [88,89]. However, conflicting evidence suggests lactate’s contribution to the pro-inflammatory response, specifically in microglia. Similar to neurons, microglia rely on OXPHOS but are metabolically reprogrammed to anaerobic glycolysis in response to mitochondrial dysfunction [90]. aSyn fibrils increase glycolytic activity, increasing lactate production and exacerbating neuroinflammation by altering microglia morphology, resulting in the production of pro-inflammatory cytokines such as interleukins and tumor necrosis factor-alpha, aggravating PD pathogenesis [91,92]. Moreover, lactate accumulation promotes AMPK signaling activation, which is associated with the apoptosis of dopaminergic neurons [93,94]. However, the complete deactivation of AMPK signaling reduced lactate production and neuronal survival, suggesting the dose-dependent role of AMPK signaling in PD pathogenesis [95]. Furthermore, lactate accumulation lowers cellular pH, resulting in lactic acidosis, whose function in the brain remains controversial. The role of lactic acidosis remains uninvestigated in neurodegeneration, but it has been shown to exacerbate neuronal damage in the N2A cell line, while lactate at low concentrations remains neuroprotective in the focal cerebral ischemia mouse model [96]. PwPD presents with a direct correlation between reduced pyruvate and increased lactate levels, mediated by LDH, which is documented to be upregulated in PD [48,80]. PDH catalyzes the conversion of lactate into pyruvate in a reaction requiring NAD^+^. However, dysfunctional NAD^+^ metabolism in PD lowers its availability for lactate-to-pyruvate conversion, suggesting that increased lactate levels may be an adaptive response to the reduced PDH activity, promoting anaerobic glycolysis in PD [97].

Despite extensive research, the ANLS model highlights the complexity and importance of lactate metabolism, yet its role in PD remains not fully understood. Evidence indicates that lactate offers both neuroprotective mechanisms via mitophagy activation and detrimental consequences via pro-inflammatory mediators. However, some phenomena, such as lactic acidosis, have not been investigated in the neurodegeneration context. The ANLS model highlights the neuron-astrocyte cooperation and interdependence in energy metabolism, emphasizing the need for improved investigation techniques, as focusing on a single cell type does not yield a comprehensive *in vivo* perspective. Therefore, the lactate’s specific role in PD should be addressed with mechanistic studies utilizing isolated neuron-astrocyte and neuron-microglia cultures, combined with nuclear magnetic resonance (NMR) analysis for metabolic measurements. This combined approach would allow the identification of lactate’s mechanism of action, as well as the role of the ANLS model in it, allowing us to leverage lactate’s neuroprotective capacity in the therapeutic development process. Although lactate is one of the most measured metabolites in PD models, specifically through proton MRSI, our knowledge of its role and detrimental consequences on disease pathogenesis remains limited, suggesting that a longitudinal research design may provide the insights that one-time-point studies have overlooked.

Additionally, transcriptomic evidence of enzyme expression alterations in glia reinforces the idea of multiple cell-type glycolytic reprogramming; however, the direction of change and underlying mechanisms are not understood. Most of the available research is based on a single-cell-type study design, predominantly neurons, which is insufficient to capture the full complexity of intercellular communication and the spectrum of metabolic interactions. Therefore, integrated experimental approaches utilizing co-cultures of neurons, astrocytes, and glia may provide mechanistic insights across various cell types and possibly explain the findings of neuronal metabolic alterations, as neurons heavily depend on astrocytic and microglial support for proper function.

## 4. Tricarboxylic acid cycle

**Figure 3.**
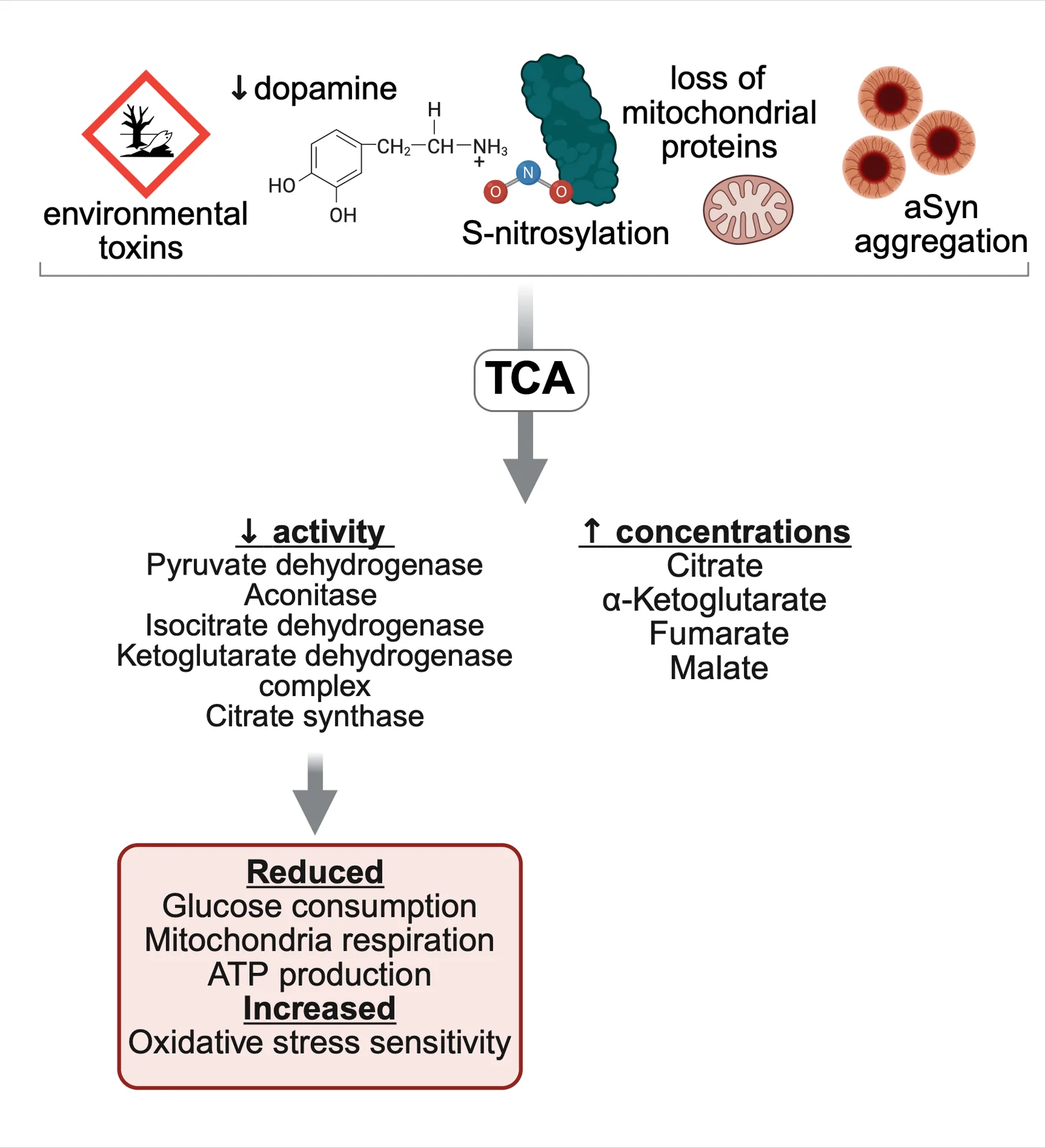
**Alterations in the tricarboxylic acid cycle in Parkinson’s disease**. This schematic illustration summarizes the metabolic alterations in the tricarboxylic acid (TCA) cycle in the context of Parkinson’s disease (PD). Multiple pathological factors modify the activity of the TCA cycle, including environmental factors, reduced dopamine availability, S-nitrosylation, loss of mitochondrial proteins, and alpha-synuclein (aSyn) aggregation. The combination of multiple pathogenic factors influences the TCA cycle flux via its effect on enzyme activity and TCA cycle intermediates’ concentrations. [100-108] Evidence across multiple PD models replicates the findings of decreased enzymatic activity in the TCA cycle, including in pyruvate dehydrogenase and ketoglutarate dehydrogenase amongst others. The reduced enzymatic activity consequently explains the accumulation of the TCA cycle intermediates such as citrate, a-ketoglutarate, fumarate, and malate. The observed changes in the TCA cycle lead to reduced glucose consumption, mitochondrial respiration, and ultimately adenosine triphosphate (ATP) production, as well as increased oxidative stress sensitivity, exacerbating PD pathogenesis and neuronal loss. Abbreviations list: aSyn: alpha-synuclein; ATP: adenosine triphosphate; PD: Parkinson’s disease; TCA: tricarboxylic acid. Figure created using BioRender.com

The TCA cycle functions to convert glycolysis-derived pyruvate into reduced forms of nicotinamide and flavin adenine dinucleotides (NADH and FADH_2,_ respectively), which act as electron donors for the ETC, driving ATP production (Figure 1). This process is significantly disrupted in PD, as evidenced by findings from *in vivo* PD models. PDH catalyzes the first gatekeeping step of the TCA; however, its activity is compromised in PD. There is an association between reduced PDH activity, lower dopamine levels in the striatum, and diminished locomotor activity in the chemically induced PD mouse model (Fig. 3) [31]. Dysfunctional PDH activity has also been observed in *DJ-1*^-/-^ mice, which exhibit dopaminergic neurodegeneration and motor impairments due to the loss of DJ-1 protein, which binds PDH to ensure its proper function [32]. Immunohistochemical analysis of post-mortem tissue from PwPD revealed its co-localization with Lewy bodies in the SNpc [33]. Consequently, PDH relocates from the mitochondria to the cytoplasm during PD pathogenesis and loses its enzymatic activity [33]. Furthermore, proton NMR (^1^H-NMR) analysis of PwPD plasma levels revealed elevated pyruvate levels, while transcriptomic analysis further reinforced decreased PDH activity in PD [34,98]. The consistent evidence of reduced PDH activity suggests TCA cycle decoupling from glycolysis, leading to elevated pyruvate levels and reduced glucose flux through oxidative metabolism. Elevated pyruvate levels are converted to lactate due to increased LDH activity in PD, signifying a metabolic shift towards anaerobic glycolysis.

aSyn aggregation is an additional factor affecting the TCA cycle functioning. For instance, in aSyn transgenic mice, aSyn fibrils bind aconitase 2 (ACO2), inhibiting its activity, leading to increased citrate and decreased isocitrate levels, further disrupting mitochondrial function [99]. Loss of ACO2 impairs mitochondrial function and autophagic flux in the *Drosophila* PD model, suggesting its role in disease progression [35]. Reduced ACO2 activity has also been observed in PwPD mononuclear cells [35]. The earliest evidence of TCA cycle dysfunction includes immunohistochemistry analysis of post-mortem tissue, suggesting impaired alpha-ketoglutarate dehydrogenase complex (KGDHC) function [36,100]. KGDHC activity is reduced by up to 50% in the cerebellum and SNpc in PwPD, correlating with impaired mitochondrial respiration, diminished ATP production, and neuronal degeneration [36,100]. KGDHC dysfunction in PD further disrupts the TCA cycle, lowering the levels of fumarate and malate while reducing glucose consumption in N2A cells and KGDHC-deficient mouse models [101]. Reduced KGDHC activity is attributed to aberrant S-nitrosylation in the *in vitro* PD model, further exacerbating energy deficits [102].

Collectively, reduced PDH, ACO2, and KGDHC activity disrupts the TCA cycle, resulting in intermediate accumulation and decreased enzymatic activity of subsequent cycle steps. MPP^+^-treated SH-SY5Y cells demonstrated reduced citrate synthase (CS) and isocitrate dehydrogenase activity (IDH) [103]. 6PPD-Q treatment of primary dopaminergic neurons has been demonstrated to reduce mitochondrial respiration, directly impacting ATP production in addition to depleting TCA cycle intermediates, including citrate, alpha-ketoglutarate, fumarate, and malate. Fang *et al.* did not specify whether the pathogenic effects of 6PPD-Q arise from the same or various mechanisms, leaving room for speculation that dysregulation of energy metabolism may be an early pathogenic event, resulting in mitochondrial dysregulation, warranting further investigation [45]. Dopamine treatment of SH-SY5Y cells decreased CS and ACO2 activity, resulting in mitochondrial dysfunction, highlighting its toxic role in disease progression. [104] IDH loss in *DJ-1*^-/-^ *Drosophila* increases oxidative stress sensitivity and mitochondrial defects in dopaminergic neurons [105]. The therapeutic potential of manipulating the TCA cycle has been investigated, demonstrating the neuroprotective capacity of ethyl pyruvate in a 6-hydroxydopamine (6-OHDA)-treated *in vitro* model [106]. Similarly, exogenous pyruvate administration rescued dopaminergic degeneration and motor impairments in MPTP-treated mice [107].

Widespread impairments in the TCA cycle are correlated with mitochondrial dysfunction and PD pathogenesis. Loss of IDH, KGDHC, and ACO2 disrupts the TCA cycle flow, decoupling it from glycolysis. The lack of available literature on the TCA cycle alterations in response to mitochondrial dysfunction in astroglia demonstrates an extensive gap in our understanding, warranting further exploration. As neurons function in a highly interconnected brain network, cell-specific TCA cycle variations may help to explain some of the observed neuronal changes and advance our knowledge of pathogenic mechanisms, revealing novel therapeutic targets for modulating cell-specific metabolic pathways.

## 5. Fatty acid β-oxidation

Twenty percent of cellular energy comes from FAO, which is a metabolic process of fatty acid (FA) activation into acyl-CoA derivatives inside the mitochondria [81,108]. The majority of FAO is believed to occur in astrocytes, given its high expression of FAO-required enzymes compared to neurons [109]. Neurons metabolize FAs poorly due to superoxide anions production, while proteomics research demonstrated more efficient mitochondria FAO in astrocytes [110,111]. Additionally, oxidation-related enzymes colocalize with astrocytes in mice and humans, supporting their role in FAO [112]. Therefore, under healthy conditions, FAO mainly occurs in astrocytes, supplying neurons with ketone bodies [113]. While available research focuses on neuronal FAO dysregulation in PD, astroglial FAO dysregulation and its contribution to PD pathogenesis require further investigation.

During neurodegeneration, energy metabolism can shift from using glucose to FAs and ketone oxidation [114]. This adaptive mechanism could take place in the early stages of PD to sustain energy production and mitigate neuronal death. Evidence linking dysregulated FAO to pathogenesis is increasing; for instance, protein analysis of post-mortem tissue revealed early-stage FAO upregulation, marked by increased expression of acetoacetyl-CoA thiolase, a key FAO enzyme [37]. Reduced mitochondrial complex I activity correlated with abnormal FAO, marked by increased plasma levels of isobutyrylcarnitine, reinforcing FAO dysregulation as a consequence of mitochondrial dysfunction [38]. Despite the evidence from some studies reporting FAO upregulation, its overall function is disrupted, as indicated by a shift from FA metabolism to lipid storage in PD. For instance, aSyn-expressing iPSCs presented with lipid accumulation, contributing to PD pathogenesis and promoting aSyn inclusions [115]. Furthermore, urinary metabolite analysis in early-stage PwPD revealed elevated furoyglycine, triglyglycine, and acyl-carnitines, suggesting FAO disruptions and lipid accumulation [116]. Acyl-carnitines transport long-chain FAs into mitochondria for FAO; hence, their accumulation indicates disrupted FAO despite successful transportation [117,118]. Disrupted long-chain FAO increases their storage as triglycerides, aggravating cell death, as evidenced by cellular studies [115,119]. Altogether, evidence points towards the upregulation of FAO to meet energy demands, but its efficiency is limited and disrupted, leading to lipid accumulation and aggravated pathogenesis.

However, evidence of increased FAO in PD remains controversial. For instance, in the *PINK1^-/-^ Drosophila* model, FAO is reduced due to ceramide accumulation, while its stimulation rescued the PD phenotype and improved ETC activity, suggesting that FAO enhancement has therapeutic potential [39]. Metabolic and trans-omic analyses of *de-novo* PwPD revealed early FAO insufficiency, suggesting its involvement in disease progression [120]. Additionally, FAO suppression marked by reduced levels of long-chain acylcarnitines and acylcarnitine-to-fatty acid ratio, has been proposed as an early diagnostic marker in PwPD [121].

FAO is an essential metabolic mechanism for ATP production, supporting energy metabolism in PD. Similarly to glycolysis, FAO appears to be differentially affected across disease stages - upregulated in early stages to meet energy demands and declining with the disease progression; however, this pattern remains controversial and not fully supported by all research available. Nevertheless, confirmed FAO disruptions divert FAs into storage in the form of triglycerides, exacerbating energy deficiency and aSyn aggregation. Extensive research demonstrates the neuroprotective potential of FAO modulation, emphasizing the need for a full understanding of PD-implicated FAO disruptions and the identification of reliable biomarkers of its dysfunction to drive the development of personalized and targeted therapeutic strategies. As already stated, similarly to glycolysis, FAO operates at varying degrees across neurons and astrocytes under healthy conditions. Astrocytes are more glycolytic, whereas the extent of astrocytic FAO in comparison to neurons is not well characterized. As reviewed elsewhere, astrocytes have the capacity for FAO, generating substrates utilized in ketogenesis, supplying neurons with ketone bodies [122]. However, the dynamics of this metabolic interaction, the transport mechanisms involved, and the neuronal utilization of ketone bodies in PwPD and PD models are overlooked, warranting further investigation. Likewise, the understanding of FAO functioning in glia and its interactions with FAO across multiple cell types highlights the scientific knowledge gap that should be addressed.

**Table 1:** Summary of *in vivo* approaches used for the investigation of brain metabolic dysregulations in Parkinson’s disease.

Investigation approach	Sample investigated	Target	Study outcome	Refs.
LC-MS	Plasma	Lipid metabolism	Identified educed frequency of hypercholesterolemia and reduced levels of triglycerides in PwPD with dementia.	[123]
		Untargeted	Discovered a significant variation in fatty acid metabolome expression between male and female PwPD.	[124]
	CSF	Lipid metabolism	Identified lipidomic signature of PwPD.	[125]
Spectrophometric assay	CSF	Lactate	Demonstrated significantly elevated levels of lactate in PwPD compared to HCs.	[86]
GC-MS	Plasma	Lactate & pyruvate	Discovered significantly elevated levels of pyruvate in PwPD compared to significantly reduced levels in patients with multiple system atrophy.	[126]
	Plasma & CSF	Untargeted	Identified disruptions in glycolysis and imbalanced energy production in PwPD compared to patients with essential tremor.	[127]
	Serum	Short-chain fatty acids	Demonstrated lower levels of propanoic, butyric and caproic acids in PwPD. Identified a correlation between propanoic acid serum levels and the severity of PwPD motor symptoms.	[128]
NMR	Serum	Untargeted	Discovered disruptions in fatty acid biosynthesis and metabolism of pyruvate in PwPD compared to HCs.	[129]
^18^FDG-PET	Frontal and parietal lobes	Glucose metabolism	Demonstrated heightened connectivity in somatomotor and frontoparietal networks in PwPD compared to HCs.	[130]
	Nucleus Basalis of Meynert		Identified a significant correlation between the atrophy of the nucleus basalis of Meynert and reduced glucose metabolism in the parietal and occipital cortices.	[131]
	Posterior and parietal cortices, cerebellum		Discovered a reduction in glucose metabolism in left posterior cortex and an increase in cerebellum glucose metabolism, 6-8 months prior the first fall episode in PwPD.	[132]
	Temporal and frontal lobes		Demonstrated glucose metabolism modification in PwPD as a result of subthalamic nucleus deep brain stimulation.	[133]
	Cerebellum, frontal lobe		Identified an association between the development of pharyngeal phase dysphagia and glucose hypermetabolism in PwPD.	[134]
	Striatum, frontal lobe		Demonstrated a positive correlation between executive functions and left cerebellar cortex metabolism.	[135]
	Thalamus, striatum, cerebellum		Demonstrated an association between glucose hypermetabolism and brain iron accumulation.	[136]

The table summarizes a range of techniques that have been used in the past five years for the investigation of brain metabolism dysregulation in PwPD. Notably, the ^1^H-MRSI has not been utilized for such investigation and, thus not included in the summary. However, the interested reader is directed towards existing reviews exploring its application in the metabolism dysregulation in PwPD. [141,142] Abbreviation list: CSF: cerebrospinal fluid; ^18^FDG-PET: ^18^Fluorodeoxyglucose positron emission tomography; GC-MS: gas chromatography-mass spectrometry; HCs: healthy controls; LC-MS: liquid chromatography-mass spectrometry; NMR: nuclear magnetic resonance; PwPD: patients with Parkinson’s disease.

## 6. *In vivo* investigation of metabolic dysregulation in Parkinson’s Disease

The vast amount of research indicating the importance of non-OXPHOS metabolic dysregulation in PD pathogenesis cannot be overlooked. Therefore, the incorporation of *in vivo* assessment approaches in PwPD could advance our understanding of the underlying mechanisms of its dysregulation, driving diagnostics improvement and therapeutics development. With that being said, currently available methods for assessing brain metabolism in PwPD remain insufficient, demonstrating the pressing need for real-time *in vivo* assessment of brain non-OXPHOS metabolism. The current gold standard approaches include metabolomics analysis and ^18^F-fluorodeoxyglucose positron emission tomography (^18^FDG-PET), with the majority of the research performed in the past five years relying on these methodologies (Table 1). Metabolomics analysis consists of the precise quantification of metabolite concentrations via biofluid sampling, yet it lacks spatial resolution and does not allow real-time assessment of metabolic changes. Moreover, metabolomics research is inherently limited in the interpretability of its findings, as identified variations in metabolites do not necessarily imply altered brain metabolism. Conversely, ^18^FDG-PET is a nuclear neuroimaging technique, utilized for the mapping of biochemical brain alterations by employing ^18^FDG to measure glucose uptake as a surrogate marker of glucose hypometabolism [137]. Similarly, ^18^FDG-PET is one of the most used techniques for assessing glucose metabolism in the PwPD, however, it offers limited (temporal and spatial) resolution and sensitivity, as well as a limited ability to identify distinct metabolic abnormalities.

Neuroimaging-based alternatives to currently available approaches include MRSI, another non-invasive approach to biochemical mapping. The underlying principle of MRSI consists of the detection of resonant frequencies of atomic nuclei, including phosphorus (^31^P-MRSI) and hydrogen (^1^H-MRSI). The study employing ^31^P-MRSI has demonstrated no significant differences in ATP concentrations between healthy controls (HCs) and PwPD, further highlighting the compensatory role of metabolic adaptations of non-OXPHOS metabolism in PwPD to maintain energy production [138]. ^1^H-MRSI has been extensively employed to detect hydrogen-containing metabolites, such as lactate, allowing the precise but indirect measurement of anaerobic glycolysis in PwPD [139]. However, recent research utilizing ^1^H-MRSI is limited, possibly due to the methodological challenges associated with the technique. Some studies report combined concentrations of lactate and lipids, as differentiating between the two presents a complex analytic process [140,141]. Furthermore, existing review articles from the 2000s indicate no significant variation in lactate levels between PwPD and HCs, either because there is no meaningful difference in lactate concentrations or the differences in lactate levels do not align with the levels detectable by ^1^H-MRSI, leading to a shift in the application of ^1^H-MRSI towards investigating other hydrogen-containing metabolites in PwPD [142]. However, both techniques indirectly assess metabolic pathways, are restricted to predefined regions of interest, and offer limited insights into glycolytic fluxes.

Another emerging approach with improved spatial resolution is chemical exchange saturation transfer (CEST), which enables the detection of metabolites with a higher spatial resolution compared to most MRSI approaches, such as lactate, at millimolar concentrations, an outcome that can’t be matched by other neuroimaging techniques available [143,144]. However, the use of CEST imaging in PwPD is currently limited. CEST imaging suffers from temporal resolution, and the signal contamination from overlapping pools of protons from various metabolites and surrounding water complicates the procedure of data analysis and interpretation [145]. However, some of CEST imaging limitations can be mitigated with the utilization of multimodal approaches, allowing the acquisition of spatially-resolved metabolic data, supporting its feasibility in clinical research [146]. ^13^Carbon-MRSI (^13^C-MRSI) is an additional promising technique, which provides higher spatial resolution compared to ^1^H-MRSI but with lower sensitivity [147]. The low natural abundance of ^13^C necessitates the exogenous ^13^C application, which easily integrates into normal metabolic processing, allowing the real-time tracking of specific metabolites, including glucose, pyruvate, and TCA cycle intermediates [148]. The low abundance can also be addressed with the hyperpolarization technique, such as dynamic nuclear polarization, aimed at increasing the signal sensitivity of low-abundant ^13^C nuclei [149]. However, the need for specialized hardware, expensive and complex workflow, and the short signal lifetimes, complicate the full implementation of this technique in clinical research [149]. Nevertheless, the feasibility of ^13^C-MRSI has already been demonstrated in early clinical research, leveraging its advantages to investigate metabolic alterations in patients with Alzheimer’s disease, chronic hepatic encephalopathy, and even healthy ageing, presenting compelling evidence for its possible application in PwPD [150-152].

## 7. Conclusion

To conclude, the well-documented mitochondrial dysfunction phenomenon in PD pathogenesis, and growing evidence support the idea of compensatory reprogramming and alterations in non-OXPHOS metabolic pathways. However, the roles and contributions of various brain cell types in these metabolic alterations in PD remain scarcely researched, underscoring an important research gap. In addition, emerging findings suggest that non-OXPHOS metabolic alterations may serve various functions, ranging from adaptive and compensatory mechanisms to pathological drivers of PD progression, which exhibit disease-stage-dependent dynamics.

Emerging research highlights the therapeutic potential of non-OXPHOS metabolism modulation, with glycolysis and FAO upregulation demonstrating neuroprotective effects. Therefore, future research should be addressed to investigate the factors contributing to the balance between compensatory, beneficial, and detrimental consequences of observed metabolic alterations and how these alterations relate to the disease stage. Such insights would not only expand our understanding of disease pathogenesis but also serve as a valuable foundation for the development of tailored and more precise therapeutic opportunities.

Furthermore, leveraging non-OXPHOS metabolic alterations as *in vivo* biomarkers of mitochondrial dysfunction should be further investigated, as growing evidence supports their causal role. Such biomarkers would quantify energy deficits in PwPD and improve disease progression tracking beyond currently available methods such as CSF analysis. Additionally, quantification of such metabolic alterations could account for individual variability, enabling personalized metabolic modulation for optimized therapeutic benefit. However, current neuroimaging techniques are limited in their ability to assess complex metabolic dysregulation in PwPD. Advancements in high-resolution imaging and multimodal integration would drive our diagnostics, disease monitoring, and therapeutic development, laying the foundation for more targeted and personalized diagnostic and treatment approaches.
